# Clinical features of retinopathy after cardiopulmonary resuscitation

**DOI:** 10.1186/s12886-023-03137-3

**Published:** 2023-09-21

**Authors:** Su Hwan Park, Sang Yoon Kim, Sung Who Park, Iksoo Byon, Seung Min Lee

**Affiliations:** 1 Department of Ophthalmology, Pusan National University Yangsan Hospital, Pusan National University School of Medicine, 50612 Yangsan, Gyeongnam Province South Korea; 2 Research Institute for Convergence of Biomedical Science and Technology, Pusan National University Yangsan Hospital, Yangsan, South Korea; 3 Department of Ophthalmology, Medical Research Institute, Pusan National University Hospital, Busan, South Korea; 4 Pusan National University School of Medicine, Yangsan, South Korea

**Keywords:** Cardiopulmonary resuscitation, Cotton wool spot, Retinal hemorrhage

## Abstract

**Purpose:**

To evaluate the clinical patterns of retinopathy in patients who received cardiopulmonary resuscitation (CPR) using wide-field fundus photography and slit-lamp fundus examination.

**Methods:**

The medical records of patients aged ≥ 18 years who survived after receiving CPR and underwent wide-field fundus photography and slit-lamp fundus examination within 3 months were retrospectively analyzed. Fundus findings, including retinal hemorrhage and cotton wool spots, were investigated. The subjects were categorized into the retinopathy and non-retinopathy groups based on the presence of fundus findings. Systemic and CPR-related factors were analyzed to compare the two groups.

**Results:**

Twenty eyes (10 patients) and 28 eyes (14 patients) were included in the retinopathy and non-retinopathy groups, respectively. The retinopathy group had longer CPR time than the non-retinopathy group (15 ± 11 min vs. 6 ± 5 min, p = 0.027). In the retinopathy group, retinal nerve fiber layer hemorrhage was observed in all eyes, and intraretinal hemorrhage was observed in 55% of the eyes. 80% of hemorrhages were located in the peripapillary or posterior pole. There were no interval changes in visual acuity, intraocular pressure, and central retinal thickness for 6 months. The average remission periods of retinal hemorrhage and cotton wool spots were 6.8 ± 2.6 month and 5.6 ± 2.1 months, respectively. No retinopathy progression was observed.

**Conclusion:**

The signs of retinopathy, such as retinal hemorrhages and cotton wool spots, which are found after CPR, mainly occur in patients who receive longer time of CPR and improve over time.

## Background

Cardiopulmonary resuscitation (CPR) is an emergency procedure performed to recover spontaneous blood circulation and breathing in patients with cardiopulmonary arrest by performing chest compression and artificial respiration to prevent irreversible hypoxic damage to the major organs. It is a life-saving technique that prevents the patient from reaching a non-resuscitable state that leads to permanent brain death [1]. The success and survival rates of patients receiving CPR has increased with the development of CPR guidelines and training programs over the past few years [2, 3]. The concerns about injuries or adverse effects caused by CPR have increased with the increase in successful CPR [4]. However, there are only few studies on ophthalmological changes after CPR [5–9, 13, 14].

Studies have reported retinal hemorrhage in pediatric patients who receive CPR [5–9]. In these studies, the causes of retinal hemorrhage include abusive head trauma, complicated systemic disease, and CPR, but cases caused by CPR are rare. Unlike pediatric studies, which require a forensic diagnosis of child abuse, the cause of cardiac arrest in adults is different [10–12]. Thus, it is necessary to study the clinical findings of the retina after CPR. However, there are only few case reports on retinopathy after CPR in adults [13, 14], and no comparative study has been conducted on retinopathy in adult patients who survive after CPR.

Therefore, this study aimed to investigate the clinical features and characteristics of retinopathy in adult patients who survived after CPR using wide-field fundus photography and fundus examination.

## Methods

From January 2014 to December 2021, we retrospectively analyzed the medical records of patients aged 18 years or older underwent ophthalmologic examination within 3 months after CPR. This study was conducted in accordance with the tenets of the Declaration of Helsinki and was conducted under the approval of the Institutional Review Board (IRB) of our hospital (IRB approval number: 05-2022-236).

Among patients who showed retinopathy, the patients with follow-up period of more than 12 months were included. Exclusion criteria were as follows: (1) media opacity including corneal opacity, cataract, uveitis, vitreous hemorrhage with invisible fundus, and others (2) past history or diagnosis of retinal disease causing retinal hemorrhage including diabetic retinopathy, retinal vascular occlusion, hypertensive retinopathy, and others (3) retinal hemorrhage caused by systemic disorder (4) abnormal retinal vascular disease such as Coats’ disease, Eales’ disease, familial exudative vitreoretinopathy, and others (5) trauma preceding the CPR. To exclude retinal hemorrhage caused by systemic disorder, (1) in cases of increased retinal hemorrhage during the follow-up period, fluorescein angiography was performed to confirm diabetic retinopathy and others (2) medical history of malignant hypertension, defined as systolic blood pressure greater than 180mmHg or diastolic blood pressure greater than 120mmHg [15], and vital records were checked (3) patients with myeloproliferative disease or leukemia were excluded if they showed Roth spots (4) anemia with hemoglobin levels below 8 g/dL were excluded [16] (5) patients with pancreatitis were excluded.

For all patients in this study, one retinal specialist (S.M.L. or I.B.) performed the slit-lamp fundus examination with a 90 diopter lens (Volk Optical, Mentor, USA) and wide-field fundus imaging (Optos California®, Optos PLC, Dunfermline, UK). Lens status was checked with slit-lamp examination. During wide-field fundus photography, the upper and lower eyelids were pulled with a cotton swab so that the widest fundus photography could be obtained. Best-corrected visual acuity (BCVA) was measured using the Snellen visual acuity chart and converted to the logarithm of the minimum angle of resolution. Intraocular pressure (IOP) was measured using a noncontact tonometer (Canon TX-20; Canon Inc., Tokyo, Japan). Central subfield macular thickness (CSMT) was analyzed using optical coherence tomography (OCT; Cirrus OCT, Carl Zeiss Inc., Dublin, CA, USA). BCVA, IOP, and CSMT were measured at baseline after CPR and after 6-month follow-up visit.

The following items were identified through electronic medical records: age, sex, medical history including underlying disease (diabetes mellitus, hypertension, chronic kidney disease, pancreatitis, cancer, connective tissue disorders and others), cause of cardiac arrest and trauma, duration from CPR to first fundus examination, CPR time, the days of stay in the intensive care unit (ICU) and use of extracorporeal membrane oxygenation (ECMO). The following laboratory tests were checked to confirm the relationship between general condition and retinal abnormalities: (1) cardiac markers including creatinine kinase (CK), CK muscle brain (CK-MB) and troponin I (2) lipid profiles including total cholesterol, triglyceride, high density lipoprotein, and low density lipoprotein (3) coagulation tests including prothrombin time, international normalized ratio, activated partial thromboplastin time and platelet count (4) D-dimer for thromboembolism (5) hemoglobin A1c (HbA1c). The results of echocardiography were investigated to confirm cardiac function factors including ejection fraction and cardiac output. CPR was performed according to regularly updated guidelines of the American college of cardiology and the American heart association [1].

The subjects were categorized into the retinopathy and non-retinopathy groups according to fundus findings. The presence of retinal hemorrhage or cotton wool spots was confirmed using wide-field fundus photography and documented fundus description. From the first fundus examination after CPR, eyes with retinal hemorrhage or cotton wool spots were classified into the retinopathy group, and eyes without these clinical features were classified into the non-retinopathy group. Retinal hemorrhage and cotton wool spots were analyzed according to location and type. The location of the retinal hemorrhage was determined using wide-field fundus photographs and categorized into peripapillary, posterior pole, and mid-periphery, according to the method described by Tandon et al. [17]. The peripapillary area was defined as the area 1 disc diameter around the disc; the posterior pole as a circle centered at the fovea, which was tangential to the nasal edge of the peripapillary area; the mid-periphery as the region outside the posterior pole up to the ampullae of the vortex veins; and the retinal periphery as the area anterior to the vortex ampullae.

The types of hemorrhage were classified as retinal nerve fiber layer (RNFL) hemorrhage, including splinter hemorrhage and Roth spots, and intraretinal hemorrhage, such as dot and blot hemorrhage, according to the depth of the hemorrhage [18].

The Statistical Package for the Social Sciences (SPSS) for Windows version 23.0 (SPSS Inc., Chicago, IL, USA) was used for statistical analyses. Normality tests were performed using the Shapiro–Wilk test. Since no factors showed a normal distribution, nonparametric tests were used. The Mann–Whitney U test was used to compare quantitative variables, including age, BCVA, IOP, CSMT, duration from CPR to fundus imaging, CPR time, cardiac markers, lipid profiles, coagulation tests, D-dimer, HbA1c, ICU stay duration, and cardiac function factors, between the retinopathy and non-retinopathy groups. Fisher’s exact test was used to compare categorical variables, including sex, lens status, underlying disease, ICU admission, and ECMO use, between the groups. Changes in BCVA, IOP, and CSMT between baseline and after 6 months were analyzed using the Wilcoxon signed-rank test. P-value < 0.05 was considered statistically significant.

## Results

Among the 47 subjects (94 eyes) who received CPR and underwent ophthalmic examination, this study included 24 patients (48 eyes) and excluded 23 patients (46 eyes) who did not meet the following study criteria: 21 patients who did not have fundus photographs taken within 3 months after CPR, 1 patient with vitreous hemorrhage, and 1 patient with uveitis. The retinopathy and non-retinopathy groups comprised 10 patients (20 eyes) and 14 patients (28 eyes), respectively.

The baseline characteristics of the patients in the two groups are summarized in Table 1. There were no significant differences between the groups in terms of baseline status, including age, sex, lens status, BCVA, IOP, CSMT, and duration from CPR to fundus imaging. Furthermore, there were no significant differences in the chronic underlying disease such as diabetes mellitus, hypertension, and chronic kidney disease, and the cause of cardiac arrest between the two groups. All patients had no medical history of trauma. Factors related to CPR, such as cardiac markers and post-CPR status, including ejection fraction, cardiac output, ICU admission, ICU stay duration, and ECMO use, were not significantly different between the two groups (Table 2). There were no significant differences in lipid profiles, coagulation tests, D-dimer, and HbA1c between the two groups. However, the CPR times of the retinopathy and non-retinopathy groups were 15 ± 11 min and 6 ± 5 min, respectively. The retinopathy group required a longer CPR time than the non-retinopathy group (p = 0.027).

**Table 1:** Baseline characteristics of the patients

Variable	Retinopathy (n = 10)	Non-retinopathy (n = 14)	P-value
Number of eyes	20	28	-
Age (years)	57 ± 13	61 ± 12	0.446
Sex (male / female)	8 (80.0%) / 2 (20.0%)	7 (50.0%) / 7 (50.0%)	0.210
Lens status (phakic / pseudophakic)	18 (90.0%) / 2 (10.0%)	20 (71.4%) / 8 (28.6%)	0.160
Underlying Disease			
Diabetes mellitus	9 (90.0%)	7 (50.0%)	0.079
Hypertension	7 (70.0%)	4 (28.6%)	0.095
Chronic kidney disease	5 (50.0%)	3 (21.4%)	0.204
Causes of cardiac arrest			
Myocardiac infarction	4 (40.0%)	5 (35.7%)	1.000
Valvular heart disease	1 (10.0%)	0 (0.0%)	0.417
Cardiomyopathy	1 (10.0%)	1 (7.1%)	1.000
Arrhythmia	4 (40.0%)	4 (28.6%)	0.673
Respiratory failure	0 (0.0%)	2 (14.3%)	0.493
Thromboembolism	0 (0.0%)	2 (14.3%)	0.493
BCVA (logMAR)	0.65 ± 0.59	0.40 ± 0.47	0.091
IOP (mmHg)	14.9 ± 3.3	14.7 ± 2.9	0.975
Central subfield macular thickness (um)	234 ± 51	245 ± 64	0.447
Duration from CPR to fundus imaging (month)	1.3 ± 0.8	1.6 ± 0.9	0.349

BCVA, best-corrected visual acuity; CPR, cardiopulmonary resuscitation; IOP, intraocular pressure

Comparison between groups using the Mann–Whitney U test or Fisher’s exact test

**Table 2:** Comparison between the retinopathy and non-retinopathy groups in CPR-related factors and post-CPR status

Variable	Retinopathy (n = 10)	Non-retinopathy (n = 14)	P-value
CPR time (min)	15 ± 11	6 ± 5	0.027*
Cardiac markers			
CK (U/L)	253.50 ± 190.58	641.00 ± 1156.88	0.850
CK-MB (ng/mL)	9.90 ± 8.42	15.05 ± 17.62	0.758
Troponin I (ng/mL)	1.51 ± 3.94	2.15 ± 3.47	0.734
Lipid profiles			
Total cholesterol (mg/dL)	176.40 ± 130.76	182.92 ± 101.90	0.522
Triglycerol (g/dL)	158.40 ± 78.89	156.85 ± 131.79	0.410
HDL (mg/dL)	42.10 ± 20.09	37.54 ± 7.23	0.976
LDL (mg/dL)	101.60 ± 113.02	91.69 ± 44.53	0.232
Coagulation tests			
PT (sec)	17.52 ± 8.37	18.39 ± 8.40	0.546
INR	1.52 ± 1.00	1.62 ± 0.99	0.546
aPTT (sec)	58.39 ± 39.11	61.63 ± 23.88	0.341
Platelet (10^3^/uL)	231.70 ± 72.02	193.14 ± 68.61	0.312
D-Dimer(ug/mL)	2.17 ± 2.14	5.36 ± 5.60	0.115
HbA1c (%)	7.54 ± 2.00	6.59 ± 2.09	0.326
Ejection fraction (%)	54.80 ± 10.75	54.23 ± 15.89	0.927
Cardiac output (L/min)	4.28 ± 0.85	3.35 ± 1.05	0.098
ICU admission (care / no care)	7 (70.0%) / 3 (30.0%)	12 (85.7%) / 2 (14.3%)	0.282
ICU stay duration (days)	7.1 ± 7.1	6.9 ± 6.8	0.966
ECMO status (apply / no apply)	3 (30.0%) / 7 (70.0%)	5 (35.7%) / 9 (64.3%)	0.763

aPTT, activated partial thromboplastin time; CPR, cardiopulmonary resuscitation; CK, creatine kinase; CK-MB, creatine kinase muscle brain; ECMO, extracorporeal membrane oxygenation; HbA1c, hemoglobin A1c; HDL, High density lipoprotein; ICU, intensive care unit; INR, international normalized ratio; LDL, Low density lipoprotein; PT, prothrombin time

Comparison between groups using the Mann–Whitney U test or Fisher’s exact test

^*^Statistically significant difference (p < 0.05)

**Fig. 1:**
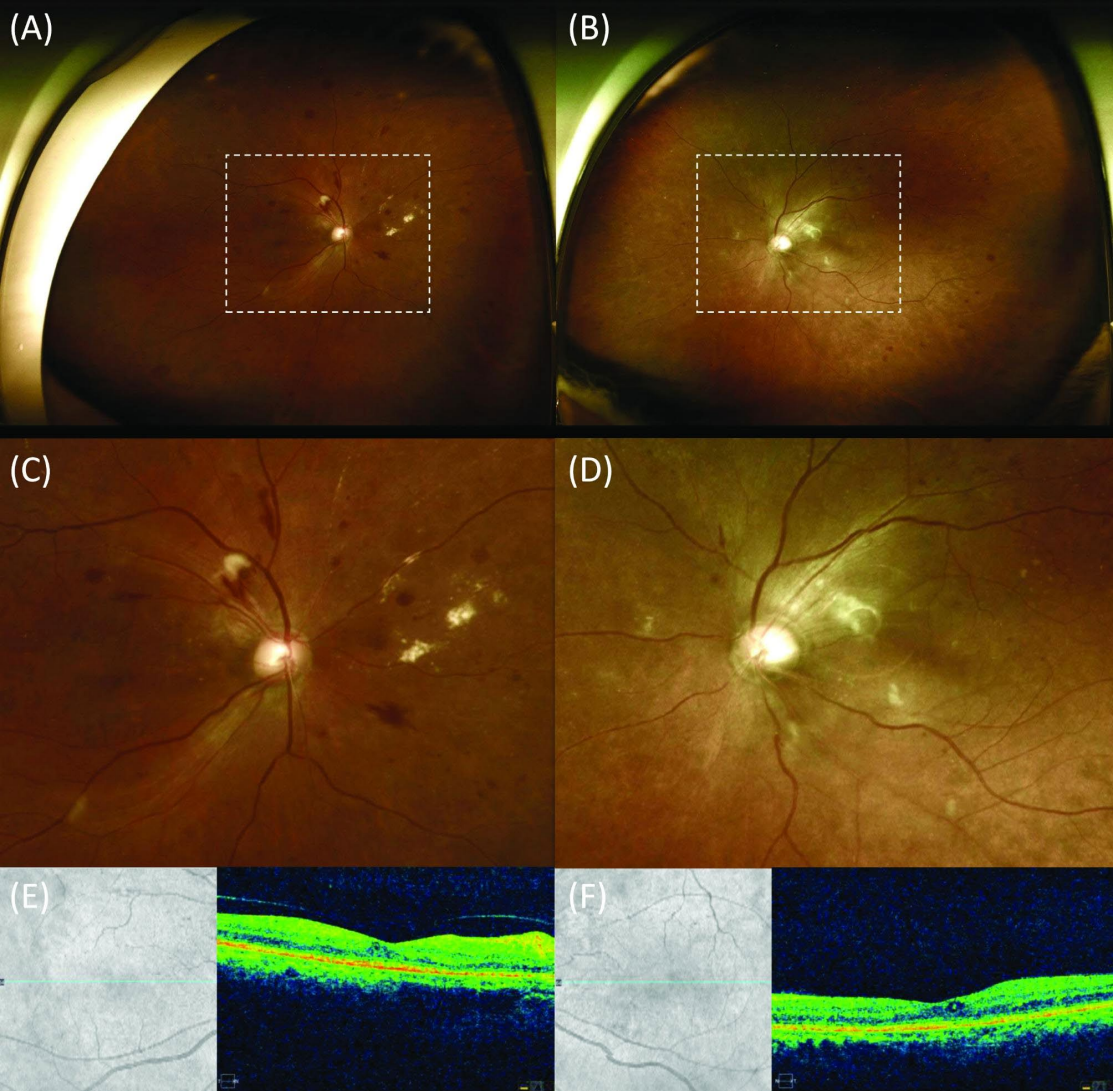
**Images of a 59-year-old female patient with retinopathy 2 weeks after cardiopulmonary resuscitation**. Wide-field fundus photography in the right (**A**) and left (**B**) eyes shows retinal hemorrhages and cotton wool spots in the posterior pole, mid-periphery, and peripapillary. (**C** and **D**) Enlarged fundus photography of white dashes rectangle images. (**E** and **F**) Corresponding optical coherence tomography in both eyes

In the retinopathy group, there were 7 eyes (35.0%) with both retinal hemorrhage and cotton wool spots, 13 eyes (65.0%) with only retinal hemorrhage, and no eyes with only cotton wool spots (Fig. 1). In the retinopathy group, all patients showed bilateral retinal hemorrhage. Among the types of retinal hemorrhage, all 20 eyes (100.0%) had intraretinal hemorrhage and 11 eyes (55.0%) had RNFL hemorrhage. Among the seven eyes with cotton wool spots, six showed both intraretinal and RNFL hemorrhages. Retinal hemorrhage of 12 eyes (60.0%) was located on the peripapillary, posterior pole, and mid-periphery, that of 4 eyes (20.0%) was located on the posterior pole and mid-periphery, and that of 4 eyes (20.0%) was located on the mid-periphery.

There were no significant differences in BCVA, IOP, and CSMT between baseline and 6 months of follow-up in the retinopathy group (Table 3).

**Table 3:** The comparison of visual acuity, IOP and macular thickness between baseline and 6 months follow-up in the retinopathy group

Variable	Baseline	6 months	P-value
BCVA (logMAR)	0.65 ± 0.59	0.59 ± 0.56	0.495
IOP (mmHg)	14.85 ± 3.25	16.71 ± 4.30	0.514
central subfield macular thickness (um)	234.39 ± 51.03	243.25 ± 37.21	0.144

CPR, cardiopulmonary resuscitation; BCVA, best-corrected visual acuity; IOP, intraocular pressure

Comparison between groups using the Wilcoxon signed-rank test

**Fig. 2:**
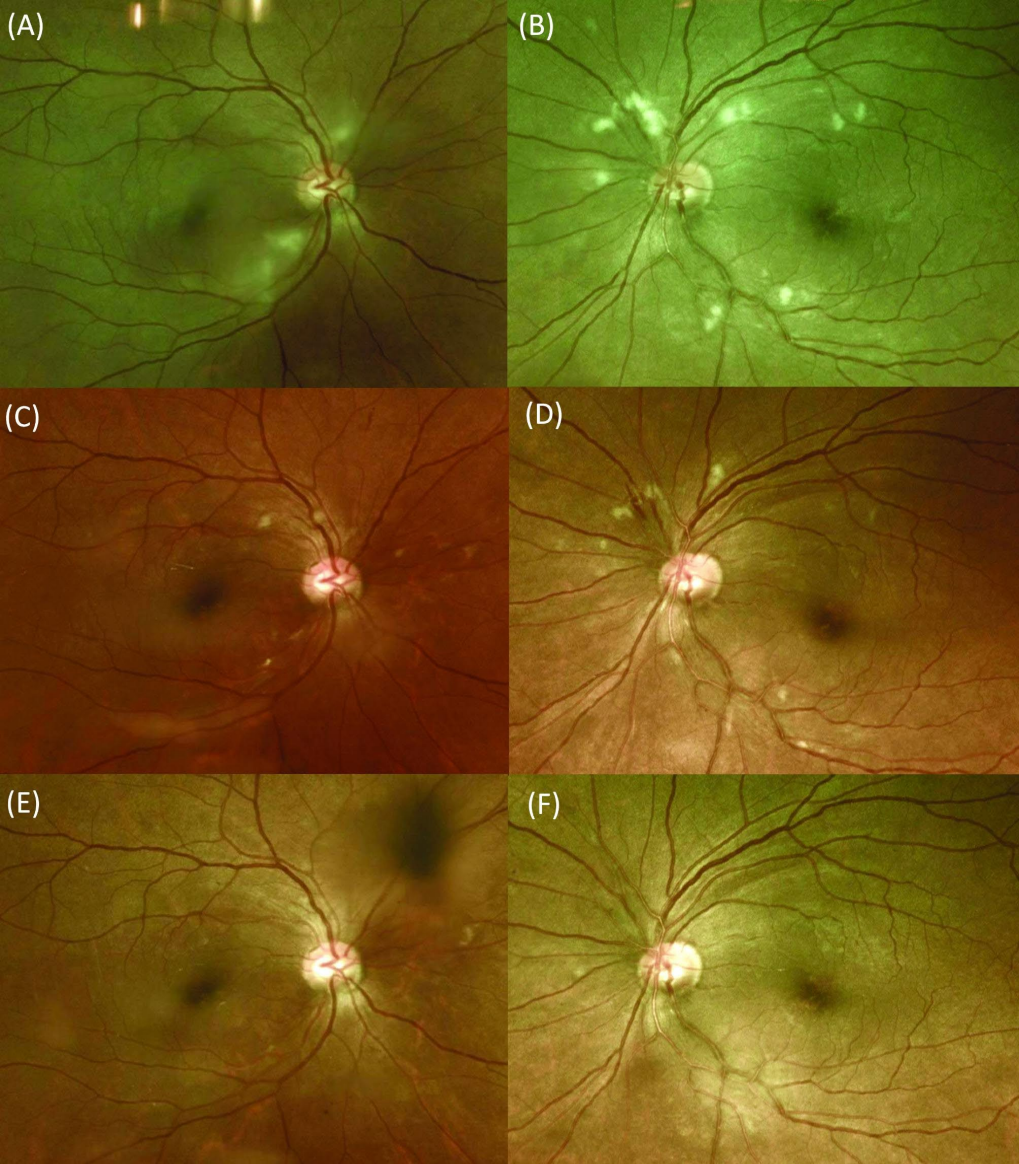
**Fundus photography in both eyes of a 45-year-old female patient with retinopathy after cardiopulmonary resuscitation (CPR)**. Cotton wool spots of the right (**A**) and left (**B**) eyes 2 weeks after CPR. (**C** and **D**) Decreasing cotton wool spots 2 months after CPR with no treatment. (**E** and **F**) Disappearance of all retinal hemorrhage and cotton wool spots 5 months after CPR

The mean follow-up period was 24 ± 21 months. In the retinopathy group, the mean self-remission periods of the retinal hemorrhage and cotton wool spots were 6.8 ± 2.6 months and 5.6 ± 2.1 months, respectively. All hemorrhages and cotton wool spots disappeared during the follow-up period, and no signs of deterioration were observed in any subject (Fig. 2).

**Fig. 3:**
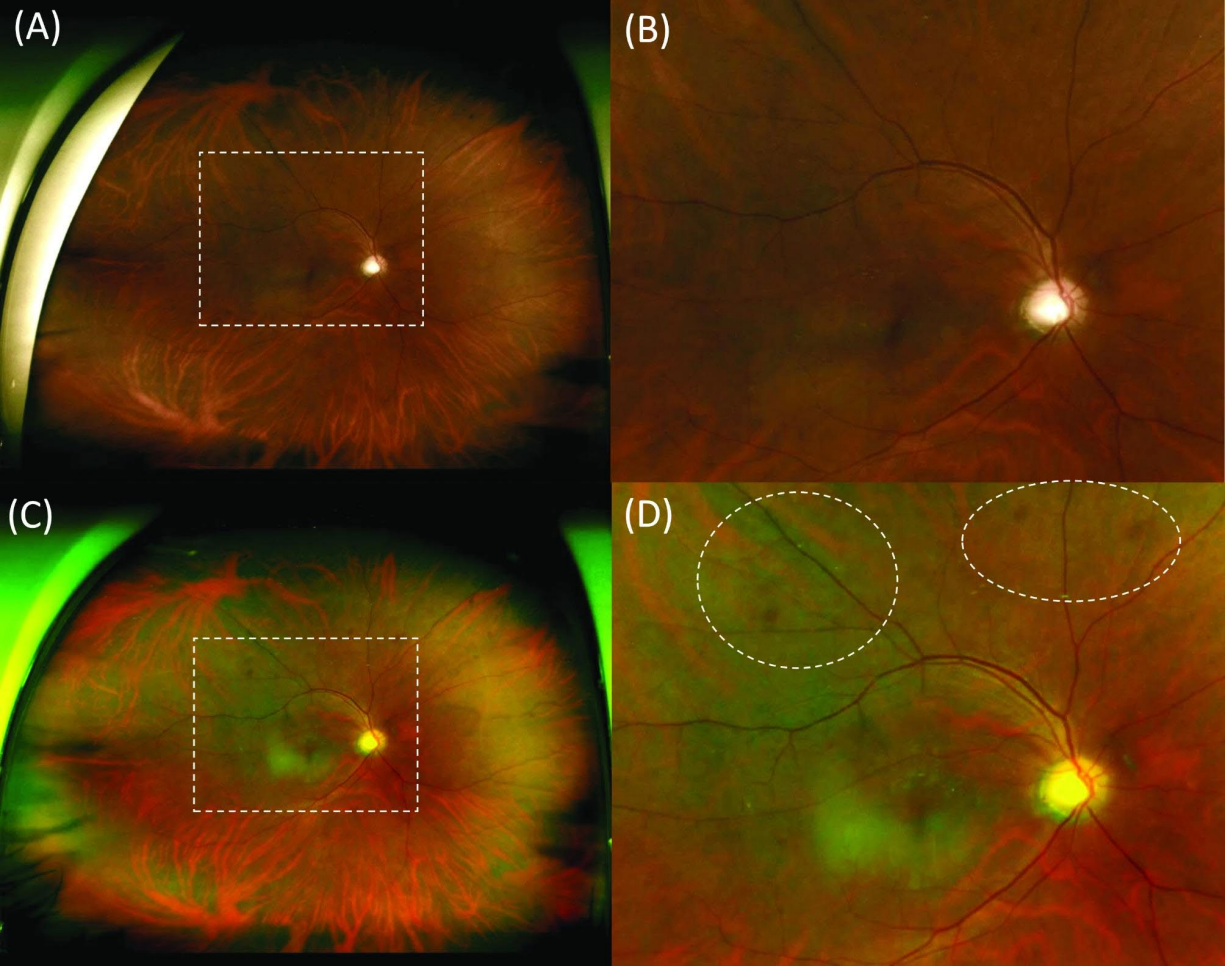
**Fundus photography in the right eye of a 50-year-old male patient before and after cardiopulmonary resuscitation (CPR)**. (**A**) There is no retinal hemorrhage or cotton wool spots 3 months before CPR. (**B**) Enlarged fundus photography of white dashes rectangle image of A. (**C**) There is retinal hemorrhage 3 months after CPR. (**D**) Enlarged fundus photography of white dashes rectangle image of C and retinal hemorrhage in white dashes circle

In 8 eyes (40.0%) in the retinopathy group with fundus findings within 12 months before CPR, retinal hemorrhage or cotton wool spots were not found before CPR (Fig. 3).

## Discussion

Our study showed that retinopathy, including retinal hemorrhage and cotton wool spots, could occur after CPR in adults, and when retinopathy occurred, the CPR time was longer than in other cases. There were no other factors that had a significant influence on the occurrence of retinopathy after CPR, including underlying disease, cause of arrest, laboratory tests and others. Retinopathy cases presented with retinal hemorrhages with or without cotton wool spots, and the main feature of retinal hemorrhages was intraretinal hemorrhage. In some cases, RNFL hemorrhage was observed. These retinal hemorrhages were observed in the mid-periphery in all cases and were distributed to the peripapillary or posterior pole in 80% of the cases. All retinal hemorrhages and cotton wool spots disappeared over time without deterioration.

Retinal hemorrhage in children receiving CPR has been documented in several studies, with an incidence rate of 11–27% [5–9]. However, according to results of these studies, when abusive head trauma was excluded, retinal hemorrhage associated with CPR was confirmed in 1 out of 43 children by Odom et al. [5], 1 out of 22 children by Pham et al. [6], and 1 out of 38 children by Binenbaum et al. [7], and the average prevalence was approximately 3.3%. Retinal hemorrhage associated with CPR in adults has been reported in only a few cases [13, 14], and its prevalence is unknown. As our study was retrospective and investigated patients who visited the ophthalmic clinic with various symptoms, it was not possible to confirm the exact prevalence. However, as 10 of the 47 (21%) patients had medical records and 10 of the 24 (42%) patients met the inclusion criteria and had no medical history of trauma, the rate of retinal hemorrhage associated with CPR may be higher than that in children.

The patterns of retinal hemorrhage associated with CPR were superficial intraretinal peripapillary hemorrhage in a report by Binenbaum et al. [7] and scattered peripapillary hemorrhage in pediatric cases according to Pham et al. [6] In adults, retinal hemorrhages present as Valsalva retinopathy in the form of peripapillary retinal hemorrhage, Terson syndrome-like appearance, and vitreous hemorrhage [13, 19, 20]. Most of the retinal hemorrhages in our study were intraretinal hemorrhages, similar to those observed in pediatric studies. However, cotton wool spots were found in one-third of the eyes with retinopathy, which was different from the results of other studies. In contrast, in other studies of pediatric and adult cases showing hemorrhages around the optic disc, the hemorrhage was mainly in the mid-periphery, and 60% of the hemorrhages were around the optic disc in our study.

The cause of retinal hemorrhage after CPR remains unclear [21, 22], but several hypotheses have been proposed. In relatively early studies, it was thought that increased thoracic pressure due to chest compression impeded intraocular venous drainage by increasing intracranial pressure through the jugular venous pressure and the paravertebral venous plexus [9, 21, 23]. As normal jugular vein valves prevent the retrograde flow of increased thoracic pressure, this hypothesis can only be applied in cases of jugular valve damage [24]. The pressures of the aorta and jugular vein bulb during CPR are 61 mmHg and 29 mmHg at peak compression and 23 mmHg and 15 mmHg at relaxation, respectively, which are lower than normal [25]. For this reason, besides increased thoracic pressure, hypoxia, Valsalva retinopathy, and Purtscher’s retinopathy have been suggested as possible mechanisms [9, 19, 21, 26]. Binenbaum et al. [7] argued that hypoxia could be more severe in out-of-hospital cardiac arrest; however, because retinal hemorrhage does not appear more frequently in out-of-hospital cardiac arrest, hypoxia is not a significant factor related to retinal hemorrhage. Valsalva retinopathy can occur when there is damage to the jugular vein valve; therefore, it is difficult to identify this as a major mechanism related to the development of retinal hemorrhage. In our study, the hemorrhage pattern did not show the characteristic findings of Valsalva, such as pre-retinal hemorrhage and sub-internal limiting membrane hemorrhage [27].

Goetting and Sowa and Weedn et al. [21, 22] reported that the cause of retinal hemorrhage observed in their study could be Purtscher’s retinopathy associated with chest compression. At that time, an increase in intrathoracic pressure due to chest compression was known to be the cause of Purtscher’s retinopathy; therefore, the mechanism was similar to previous theories. Purtscher’s retinopathy is an occlusive microvasculopathy that develops after trauma and presents with retinal findings, including cotton wool spots, retinal hemorrhages, and Purtscher’s flecken [28, 29]. Fundus findings in this study showed lesions similar to those of Purtscher’s retinopathy, such as superficial to intraretinal hemorrhages and cotton wool spots, although there were no Purtscher’s flecken. In a systematic review, Purtscher’s flecken was present in only approximately 60% of patients, and in the study by Agrawal and McKibbin, Purtscher’s flecken was found in 3 out of 10 patients who received chest compression [28, 29]. For this reason, although there was no Purtscher’s flecken, it is possible that the retinopathy in our study was caused by the same mechanism as that of Purtscher’s retinopathy caused by chest compression during CPR.

It has recently been accepted that the pathogenesis of Purtscher’s retinopathy is due to microembolization followed by arteriolar precapillary occlusion [28]. After manual CPR, pulmonary bone marrow embolism and fat embolism were found in 26.7% and 20% of patients, respectively, and pulmonary bone marrow embolism was correlated with CPR time [30]. Because the fat embolus is relatively small and easily deformable, it can move to the retina through the foramen ovale (present in 20–25% of adults), lung capillaries, or arteriovenous shunts around the lungs [31]. Occlusion of the precapillary arteriole by an intermediate-sized embolus causes Purtscher’s flecken. However, in the case of a small fat embolus, obstruction of the retinal capillary can cause cotton wool spots or retinal hemorrhage [32]. For this reason, it seems that the appearance of Purtscher’s retinopathy was caused by fat emboli derived from the rip or sternum caused by chest compression in patients who received a long CPR time in our study. Agrawal and McKibbin [32] reported the disappearance of cotton wool spots, retinal hemorrhage, and Purtscher flecken at 6 months in Purtscher’s retinopathy, whereas in our study, retinal hemorrhage and cotton wool spots disappeared at 5.6 months, showing similar results.

In our study, 9 of 10 patients in the retinopathy group had diabetes mellitus, which was higher than the 27–31% reported in other studies (binomial distribution test, p = 0.002) [33, 34]. As fundus findings, including microaneurysm, hard exudate, retinal swelling, venous beading, and neovascularization in diabetic retinopathy, except dot-blot hemorrhage and cotton wool spot, were not observed and abnormal findings gradually disappeared during follow-up, it is assumed that the possibility of diabetic retinopathy is low. In addition, in 4 of the 10 patients with fundus findings within 12 months before CPR, diabetic retinopathy was not found before CPR.

This study has several limitations. This was a retrospective, small-case study. In terms of patient selection, there was a selection bias because most patients were referred for treatment because of complaints of ophthalmic discomfort. In addition, the underlying diseases of patients and those that cause CPR were diverse. In terms of patient examination, the initial condition of the patients was not stable, and there were limitations in performing fundus examination in all patients. To establish differential diagnosis from other retinal diseases, including diabetic, hypertensive retinopathy, anemic retinopathy, and others and to assess the retinal anatomic and vascular status, ophthalmic examinations, such as OCT, OCT angiography, and fundus angiography, were required. However, these had not been performed in several patients. Patients with systemic conditions that could commonly cause retinal hemorrhages were excluded, cases with retinopathy due to systemic complication were excluded by additional examination and medical record check, and laboratory tests were checked to identify potential confounding factors that could cause retinal hemorrhage. Additionally, in all included retinopathy patients, the findings of retinopathy had improved over time, suggesting that the effects of other retinal diseases were minimal.

In conclusion, as the success rate of CPR increases and its practice becomes more common [35, 36], it is becoming more likely that fundus examinations will be performed in patients who have received CPR. If a retinal hemorrhage is found in these patients, it is necessary to differentiate it from other retinal diseases. In cases of intraretinal hemorrhage with or without RNFL hemorrhage or cotton wool spots after CPR, retinopathy associated with CPR can be suspected if there are no characteristic findings of other retinal diseases. Retinopathy appears when CPR is prolonged and disappears over time.

## Data Availability

All data generated or analyzed during this study are included in this article. Further inquiries can be directed to the corresponding author.

## List of Abbreviations

BCVA: Best-corrected visual acuity
CK: Creatine kinase
CK-MB: Creatine kinase muscle brain
CPR: Cardiopulmonary resuscitation
CSMT: Central subfield macular thickness
ECMO: Extracorporeal membrane oxygenation
HbA1c: Hemoglobin A1c
ICU: Intensive care unit
IOP: Intraocular pressure
OCT: Optical coherence tomography
RNFL: Retinal nerve fiber layer
